# Antagonistic Activity and Molecular Insights into the Biocontrol of Korla Pear Fire Blight by *Paenibacillus* sp. TRMB57781

**DOI:** 10.3390/biology15100764

**Published:** 2026-05-11

**Authors:** Xirui Wang, Chaowen Liu, Jinduo Zhao, Yuxin Si, Zagipa Sapakhova, Jiangtao Gao, Xiaoxia Luo

**Affiliations:** 1 College of Life Science, Tarim University, Alar 843300, China; 18589042217@163.com (X.W.); 15593538060@163.com (C.L.);; 2 State Key Laboratory of Ecological Pest Control for Fujian and Taiwan Crops, College of Life Sciences, Fujian Agriculture and Forestry University, Fuzhou 350002, China; 3 Institute of Plant Biology and Biotechnology, Almaty 050040, Kazakhstan

**Keywords:** *Erwinia amylovora*, *Paenibacillus*, Korla fragrant pear, biological control, secondary metabolites, whole genome, biocontrol mechanism

## Abstract

Pear fire blight caused by *Erwinia amylovora* necessitates environmentally friendly biocontrol alternatives. Strain TRMB57781, isolated from Alar chemical plant wastewater, was identified as *Paenibacillus* sp. via polyphasic taxonomy and 16S rRNA gene analysis. Whole-genome sequencing revealed a biosynthetic gene cluster for fusaricidin B. Fermentation medium optimization with glucose and cottonseed meal significantly enhanced antibacterial activity. In vitro assays demonstrated that a 1:50 dilution of the fermentation broth achieved 84.62% control efficacy on fragrant pear inflorescences, markedly exceeding agricultural streptomycin (69.48%). Comparable efficacy to streptomycin was observed in detached leaf assays and potted plant protective and curative trials. Through HR-TOF MS analysis combined with genomic verification, the active compound was identified as a key antimicrobial metabolite associated with the antagonism against *E. amylovora*. This study presents *Paenibacillus* sp. TRMB57781 as a promising biocontrol agent and provides genomic and metabolomic insights into its antibacterial activity against pear fire blight.

## Simple Summary

Fire blight is a serious bacterial disease that can quickly kill the blossoms and branches of pear trees, sometimes destroying entire orchards in a single season. Currently, farmers rely heavily on chemical sprays, such as streptomycin, to control the disease, but these treatments can harm the environment and lead to resistant bacteria. This study explored a natural and safer alternative. We discovered a special bacterium, *Paenibacillus* sp. strain TRMB57781, living in the wastewater of a chemical plant. Surprisingly, this microbe produces a natural antibiotic compound that kills the fire blight pathogen without harming the pear tree. In laboratory tests on pear flowers and leaves, a diluted solution of this beneficial bacterium provided better protection than the traditional chemical streptomycin. Even when the disease was already present, the bacterium helped the plant recover as effectively as the chemical treatment. These findings offer a promising, environmentally friendly way to protect valuable pear crops. By using this natural soil bacterium instead of synthetic chemicals, growers can reduce pollution and help preserve the health of both the orchard ecosystem and the consumers who enjoy the fruit.

## 1. Introduction

Pear fire blight is a highly destructive bacterial disease caused by *Erwinia amylovora*. First discovered in North America in the 19th century, it has spread rapidly and is now widely distributed across multiple countries and regions in Europe, Asia, Africa, South America, and Oceania [1,2], posing a severe threat to *Pyrus* species and numerous Rosaceae plants and causing significant economic losses [3]. China is an important place of origin for *Pyrus* plants [4] and one of the world’s top three pear-producing countries, with the pear industry making remarkable contributions to the agricultural economy. Among them, Korla fragrant pear, originating from southern Xinjiang, is a national geographical indication product of China, and its related income accounts for over 30% of the per capita net income of local farmers and herders. However, in recent years, pear fire blight has also emerged in Korla fragrant pear planting areas in southern Xinjiang [5]. The outbreak of this disease not only leads to a sharp drop in fruit yield in the current season but also exerts long-term negative impacts on the overall vigor and lifespan of fruit trees [6], directly threatening the stable development of the fragrant pear industry. This pathogen has a wide host range and clear transmission pathway, mainly infecting the leaves, flowers, fruits, buds, branches, trunks, and roots of fragrant pears. In naturally infected plants in the field, flowers are the first to show symptoms [7], which appear after flowering in April. The pathogen spreads from the initially infected organs to perennial branches [8], and can further spread to the trunk and roots, ultimately leading to the death of the host plant [9].

Traditionally, the control of pear fire blight has relied primarily on chemical fungicides such as streptomycin and oxytetracycline [10,11]. Currently, agricultural streptomycin exhibits significant inhibitory effects against the pathogen, with a control efficiency ranging from 60% to 97%, and the bactericidal efficiency of kasugamycin is comparable. While chemical control can effectively inhibit the growth and spread of the pathogen in the short term, the long-term and excessive use of chemical agents has brought about a series of serious problems [12]. Firstly, the pathogen’s resistance to chemical fungicides is constantly strengthening [13], resulting in a gradual decline in control efficacy. This necessitates continuous increases in dosage or the use of new agents [14], further raising control costs and difficulties. Secondly, residues of chemical agents can contaminate soil, water bodies, and other ecological environments, disrupting the ecological balance and affecting the survival and reproduction of non-target organisms [15,16]. Thirdly, high-dose chemical residues may also endanger human health, posing potential food safety risks. Therefore, exploring safe, efficient, and sustainable biological control methods to replace or partially substitute chemical control has become a research hotspot and urgent need in the field of pear fire blight management. Bacteria of the genus Bacillus have demonstrated enormous potential in the biological control of plant diseases due to their unique biological characteristics [17,18]. *Bacillus* species can produce a variety of antibacterial substances [19], such as lipopeptides (e.g., fengycin) [20,21], proteins [22], lipids [23], and polysaccharides [24,25]. These substances exert antibacterial effects by inhibiting pathogen growth, interfering with metabolic processes, and damaging cell walls or membranes. Meanwhile, *Bacillus* possesses excellent colonization ability, enabling it to rapidly colonize plant rhizospheres, surfaces, and other sites, form dominant microbial communities, and compete with pathogens for nutrients and ecological niches, thereby suppressing pathogen invasion [26]. Additionally, *Bacillus* can secrete various plant growth regulators during metabolism [27], such as auxins, cytokinins, and gibberellins, which promote plant growth and development and enhance stress resistance [28]. Coupled with advantages such as ease of cultivation, low fermentation costs, and environmental friendliness, *Bacillus* has become a key research focus and popular biocontrol resource in the field of biological control.

Bacteria of the genus *Paenibacillus* have demonstrated enormous potential in the biological control of plant diseases. *Paenibacillus* species can antagonize pathogens through multiple mechanisms, such as secreting antimicrobial metabolites, inhibiting mycelial growth and spore germination, and inducing host systemic resistance. For instance, *P. polymyxa* SY42 and *P. polymyxa* GRY-11 have been shown to significantly inhibit *Fusarium* spp., which cause Atractylodes chinensis root rot and apple replant disease, respectively [29,30]. Genomic analyses have further revealed that the fusaricidin B biosynthetic gene cluster is present in *Paenibacillus* strains from diverse habitats, including lzh-N1 from pear rhizosphere, HOB6 from hemp seed oil, and J2-4 from ginger rhizosphere, suggesting that fusaricidin-type lipopeptides may be a key molecular basis for their biocontrol activity [31,32,33,34]. Mechanistically, studies have shown that fusaricidins primarily disrupt bacterial membrane ion transport systems, causing leakage of intracellular nucleic acids and proteins, while also inhibiting spore germination and hyphal growth [35].

In this study, a *Paenibacillus* strain TRMB57781 with strong antibacterial activity against *E. amylovora* was isolated from wastewater of Alar Chemical Plant. Its taxonomic status was clarified using polyphasic taxonomic identification, and its biocontrol potential against pear fire blight was systematically explored. This research aims to provide new biocontrol strain resources and theoretical basis for the biological control of pear fire blight, promote the popularization and application of biological control technology in the pear industry, reduce the use of chemical pesticides, and realize the green and sustainable development of the pear industry.

## 2. Materials and Methods

### 2.1. Activation of Pathogenic Bacteria and Test Strains

*E. amylovora* and strain TRMB57781 were both preserved in the strain collection room of Key Laboratory of Biological Resources Protection and Utilization in Tarim Basin, Xinjiang Production and Construction Corps (stored in −80 °C frozen glycerol stocks). For activation, the frozen strains were taken and aseptically inoculated onto 90 mm Petri dishes containing Lysogeny Broth (LB) solid medium; both *E. amylovora* and TRMB57781 were incubated at 37 °C for 24–48 h. After single colonies formed, a single colony was picked and inoculated into 50 mL LB liquid medium (in a 250 mL Erlenmeyer flask), followed by shaking culture at 37 °C and 120 rpm for 24–48 h to obtain bacterial suspensions in the logarithmic growth phase.

### 2.2. Polyphasic Taxonomic Identification and Whole-Genome Sequencing Analysis

The taxonomic identification of the strain was performed using a polyphasic taxonomic approach. Morphological observations included recording colony characteristics, and scanning electron microscopy (SEM) (Thermo Fisher Scientific, Waltham, MA, USA) was used to analyze surface ultrastructure. Physiological and biochemical tests comprised the catalase test, with the production of bubbles in freshly prepared 3% H_2_O_2_ solution as the criterion for a positive result; the oxidase test was conducted using API test strips (API-NE, API-50CHB, and API-ZYM); and carbon-nitrogen source metabolic profiles were analyzed via the BIOLOG system (bioMérieux SA, Marcy-l′Étoile, France). For molecular identification, genomic DNA was extracted using the CTAB method, and the 16S rRNA gene fragment was amplified with primers 27F (5′-AGAGTTTGATCCTGGCTCAG-3′) and 1492R (5′-TACGGCTACCTTGTTACGACTT-3′). The amplified products were bidirectionally sequenced by Sangon Biotech (Shanghai) Co., Ltd. (Shanghai, China), and then aligned with high-similarity sequences in the EzBioCloud (https://www.ezbiocloud.net/, accessed on 1 March 2026) database. Phylogenetic relationships were calibrated using 24 reference strains and the type strain *Escherichia coli* K-12 MG1655^T^ as an outgroup. A phylogenetic tree was constructed using the maximum likelihood method in MEGA X software (v10.2.6), with 1000 bootstrap replications to verify node reliability.

Whole-genome analysis was conducted based on next-generation sequencing (NGS) technology. A single colony was picked and inoculated into TSB liquid medium (from Beijing Aoboxing Biotechnology Co., Ltd. (Beijing, China)) containing 17 g/L tryptone, 5 g/L papaic digest of soybean meal, 5 g/L sodium chloride, 2.5 g/L glucose monohydrate, and 2.5 g/L dipotassium hydrogen phosphate (pH 7.2–7.5). After shaking culture at 37 °C and 120 r/min in a constant-temperature shaker for 24 h, bacterial cells were collected by centrifugation at 8000 r/min. Whole-genome sequencing, splicing, assembly, and annotation of TRM57781 were performed by Nanjing Personalbio Technology Co., Ltd. (Nanjing, China) using the Illumina HiSeq 2000 sequencing platform (Illumina Inc., San Diego, CA, USA).

Ten type strains with the highest 16S rRNA sequence similarity to TRMB57781 were selected via NCBI-BLAST (National Center for Biotechnology Information, https://www.ncbi.nlm.nih.gov, accessed on 1 March 2026) alignment, and their whole-genome reference sequences were downloaded for pan-genome difference analysis. Meanwhile, antiSMASH 8.00 [36] was used to predict biosynthetic gene clusters of TRMB57781, and IPGA [37] was applied for comparative genomics analysis. Genome assembly was performed using SPAdes (v3.15.5) [38], and genome annotation was carried out with Bakta v1.8.2 (database version v5.0, light mode) to identify functional elements such as CDSs, tRNA, and rRNA. Antibiotic resistance-related features were predicted using the CARD RGI tool integrated in the Proksee [39] platform (https://proksee.ca/, accessed on 1 March 2026) to analyze the genomic potential for drug resistance.

### 2.3. Antimicrobial Activity and Fermentation Component Optimization of TRMB57781

The antimicrobial efficacy of TRMB57781 against *E. amylovora* was assessed through the agar well diffusion method. A single colony of *E. amylovora* was picked and inoculated into LB liquid medium, followed by cultivation at 37 °C and 180 r/min until OD_600_ = 0.5. Subsequently, 100 μL of the bacterial suspension was evenly distributed on the surface of LB plates using a sterile spreader. Sterile 6 mm diameter punchers were employed to create holes on plates containing pathogens, into which 200 μL of TRMB57781 fermentation broth was introduced. Following a 24-h incubation period at 37 °C, the diameter of the resulting inhibition zone was measured to preliminarily assess the antimicrobial efficacy. To screen high-yield media, experiments were conducted based on antagonistic activity.

To evaluate high-yield media, experiments were conducted based on antagonistic activity. The experimental groups consisted of TSB, N1 (yeast extract 6 g/L, fructose 10 g/L, soybean meal 3 g/L, MgSO_4_ 0.20 g/L, pH 7.2–7.5, sterilized at 121 °C for 30 min), N2 (corn flour 13.74 g/L, fish meal 15.00 g/L, yeast extract 15.73 g/L, corn steep liquor 5.00 g/L, MgSO_4_ 0.59 g/L, KH_2_PO_4_ 0.30 g/L, pH 7.0–7.6, sterilized at 121 °C for 30 min), and N3 (glucose 24.27 g/L, soybean meal 29.05 g/L, beef extract 3 g/L, NH_4_NO_3_ 4 g/L, L-leucine 2.3 g/L, KH_2_PO_4_ 3 g/L, NaCl 3 g/L, FeSO_4_ 0.02 g/L, sterilized at 121 °C for 30 min), with LB medium serving as the control group. During the experiment, 10 mL of each medium was dispensed into sterilized glass sample bottles. Following inoculation with TRMB57781, the cultures were incubated at 37 °C with shaking at 180 r/min for 72 h. Subsequently, the pathogen suspension was spread on plates, holes were made, and 200 μL of fermentation broth was injected into each hole. Following incubation at 37 °C for 24 h, the inhibition zone diameters were measured. The mean diameter was determined from three independent biological replicates.

During the carbon source optimization phase, the basal medium’s glucose was substituted with equivalent quantities of cellulose, xylose, and fructose. Subsequently, in the nitrogen source optimization phase, the initial soybean meal was exchanged with equal proportions of cottonseed meal, corn steep liquor powder, and peanut meal. After incubation at 37 °C and 180 rpm for 60 h, the inhibition zone diameters were measured as described above, and the most effective carbon–nitrogen source combination was selected. Subsequently, the fermentation medium system was established.

The seed culture was prepared in LB liquid medium by incubating at 37 °C with shaking at 180 r/min for 24 h to yield 300 mL of seed culture. The optimal fermentation broth was prepared using N3S medium (glucose 24.27 g/L, cottonseed meal 29.05 g/L, beef extract 3 g/L, NH_4_NO_3_ 4 g/L, L-leucine 2.3 g/L, KH_2_PO_4_ 3 g/L, NaCl 3 g/L, FeSO_4_ 0.02 g/L) and sterilized at 121 °C for 30 min. Large-scale fermentation was performed in a 100-L stirred-tank fermenter with a working volume of 30 L of N3S medium. After sterilization, 2% seed culture was aseptically inoculated. The fermentation was conducted at 37 °C for 6 days. Temperature was monitored and maintained at 37 °C using a temperature probe. Agitation was provided by turbine impellers at 180 rpm. The pH was allowed to vary naturally (initial pH was approximately 7.0–7.5). Filter-sterilized compressed air was supplied at a flow rate of 1.0 vvm. Dissolved oxygen was not monitored during the fermentation.

All antagonistic activity assays were performed with three independent biological replicates (independently grown cultures), and results are expressed as mean ± standard deviation of the three biological replicates.

### 2.4. Evaluation of the Biocontrol Potential of TRMB57781 Against Fragrant Pear Fire Blight

To systematically evaluate the biocontrol potential of TRMB57781, this experiment employed fragrant pear branches with flower buds from the orchard of Tarim University, healthy and disease-free leaves, and greenhouse-cultivated Korla fragrant pear potted seedlings as test materials, and treatments were carried out using the prepared TRMB57781 fermentation broth. The specific experimental methods are as follows.

#### 2.4.1. Inflorescence Preventive Treatment

Fragrant pear branches with flower buds were selected and subjected to hydroponic culture in tissue culture bottles using a sterile aqueous solution containing 3% sucrose in the laboratory. After all flowers bloomed completely, undiluted TRMB57781 fermentation broth was inoculated onto the inflorescences by spraying to ensure full wetting. The treated inflorescences were then incubated for 24 h under culture conditions of 25 °C and 75% relative humidity, followed by inoculation with the pathogen at a concentration of 1 × 10^9^ cfu/mL via spraying.

#### 2.4.2. Leaf Curative Treatment

Healthy, disease-free and uniform-sized fragrant pear leaves were picked from the orchard. In a laminar flow hood, the leaf surfaces were punctured three times with a sterile bamboo stick, and the pathogen was inoculated. The leaves were then placed in a constant temperature incubator at 30 °C for cultivation. After disease symptoms appeared on the leaves, TRMB57781 fermentation broth was applied using a cotton swab, and the disease development was dynamically observed.

#### 2.4.3. Potted Seedling Treatment

From March to April 2024, 50 healthy and pest-free annual Korla fragrant pear seedlings purchased from the local market were planted in the greenhouse of the South Xinjiang Academy of Modern Agriculture, Tarim University, Alar, Xinjiang. The cultivation containers were flower pots with a height of 20 cm and an inner diameter of 16 cm. Routine management included thorough watering once every 7 days and compound fertilizer application once a month. Before the experiment, the seedlings were pruned to achieve a basically uniform plant height. When the new shoots grew to 10–20 cm in length, excessively long and short branches were removed 5 days prior to pathogen inoculation, and current-year branches with uniform growth were retained. The seedlings were divided into a prevention group and a treatment group: for the prevention group, TRMB57781 fermentation broth was sprayed first, and the pathogen at a concentration of 1 × 10^9^ cfu/mL was inoculated 48 h later, followed by wrapping with plastic wrap for moisture retention; for the treatment group, the pathogen was inoculated first, and the seedlings were wrapped with plastic wrap for 48 h to retain moisture before spraying with the fermentation broth.

All experiments were set up with control treatments, which were as follows: the negative control was treated with fresh sterile N3S culture medium instead of TRMB57781 fermentation broth, with all other treatments kept consistent; the positive controls included 72% agricultural streptomycin sulfate soluble powder sprayed at the dosage specified in the instruction manual, and diluted solutions of TRMB57781 fermentation broth prepared with sterile water at ratios of 1:50, 1:100, and 1:500.

For the inflorescence experiment, each group contained 20 flowers with 3 replicates, and the experimental results were observed 3 days after treatment. For the leaf experiment, the disease status was dynamically recorded at 1, 3, and 5 days post-inoculation, and the lesion area was statistically analyzed. For the potted seedling experiment, 3 replicates were set with 10 seedlings per group. The control efficacy of the prevention group was observed at 2, 7, 14, and 21 days after application; the therapeutic effect during the latent period of the treatment group was determined at 2, 7, 14, and 21 days after spraying the fermentation broth. The observation indicators for all groups included the total number of branches, the number of diseased branches, and the proportion of lesion length to the total branch length, and the treatment group additionally recorded the therapeutic effect during the latent period.

### 2.5. Isolation and Purification of Active Metabolites Derived from TRMB57781

Strain TRMB57781 was cultured in LB liquid medium with shaking for 48 h until reaching an OD_600_ value of 0.8–1.0. Subsequently, three distinct extraction methods were employed to procure crude extracts of antagonistic substances, followed by the evaluation of their biological activities.

The hydrochloric acid precipitation method involved adjusting 150 mL of sterilized fermentation filtrate to pH 2.0 with 12 mol/L HCl. The solution was then incubated at 4 °C for 12 h, followed by then centrifugation at 12,000 r/min at 4 °C for 20 min. Subsequently, the precipitate obtained was dissolved in sterile water to yield the crude isolatelipopeptide extract.

Ammonium sulfate was gradually introduced to the sterilized filtrate until saturation was reached in the method of ammonium sulfate saturation precipitation. This process selectively precipitated proteins by disrupting the hydration shell due to the elevated salt concentration. Following a 12 h incubation at 4 °C, the mixture underwent was centrifugation at the same temperature for 20 min. Subsequently, the precipitate was harvested, and yielded the crude protein extract.

In the organic solvent extraction procedure, the filtrate was combined with a mixed solvent of methanol and ethyl acetate in a 1:3 ratio. The mixture was subjected to ultrasonic extraction for 4 h, and the upper layer was then retrieved. Following concentration using rotary evaporation, the resulting extract was dissolved to isolate medium-polar and non-polar compounds.

Activity evaluation was conducted through the plate punching method, wherein holes were created on LB plates coated with *E. amylovora*, 200 μL of crude extract treated with a sterile filter membrane was applied, and the diameter of the inhibition zone was determined following an incubation period at 37 °C for 4 days.

Analysis of the extraction and purification of lipopeptide active substances was conducted utilizing ultra-performance liquid chromatography-quadrupole time-of-flight mass spectrometry (UPLC-Q-TOF MS). The ACQUITY UPLC BEH C18 column (2.1 mm × 50 mm, 1.7 μm) was utilized for chromatography. The mobile phase consisted of 0.1% formic acid in water (A) and methanol (B) at a ratio of 10:90. The flow rate was set at 0.6 mL·min^−1^, with a column temperature of 30 °C and an injection volume ranging from 0.1 to 1.0 μL. Mass spectrometry utilized an electrospray ionization (ESI) source, scanning *m*/*z* range of 100–1500 in positive ion detection mode. Parameters included capillary voltage of 3.0 kV (positive ions) and 2.5 kV (negative ions), nozzle voltage of 40 V, dry gas flow rate of 800 L/h, ion source temperature of 120 °C, argon collision gas, and collision energy of 20–50 eV. The collected experimental data were calibrated in real time using Lock Mass and i-FIT analysis.

### 2.6. Data Processing and Analysis

After preliminary organization of experimental data using Excel, statistical analysis was performed with SPSS 24.0 software, with *p* < 0.05 set as the threshold for determining significant differences. The significance test of differences was conducted using Duncan’s multiple range test. Graph plotting was completed using GraphPad Prism 8.0 and Origin 2024 software.

## 3. Results

### 3.1. Polyphasic Taxonomic Identification Was Conducted on Strain TRMB57781

**Figure 1:**
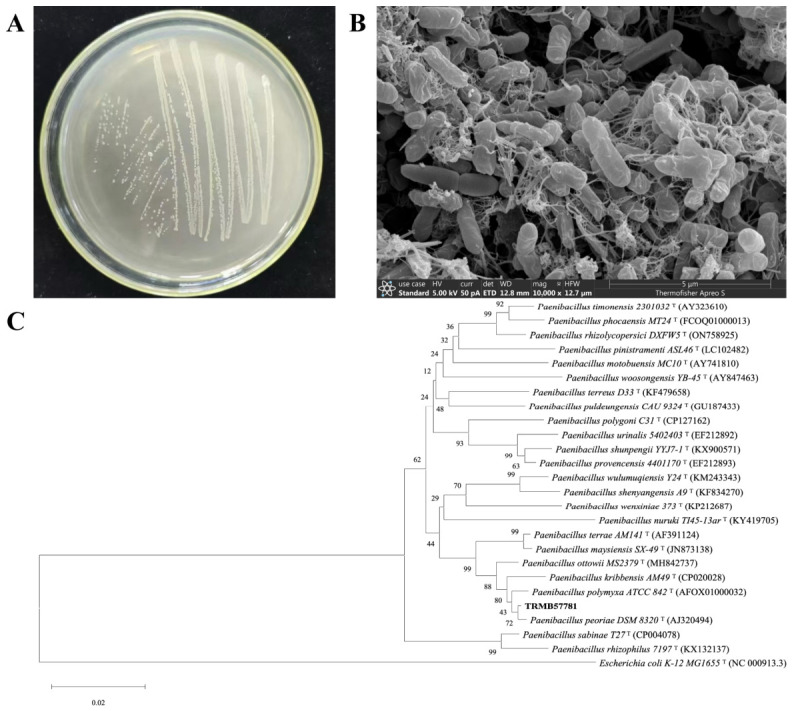
**Polyphasic taxonomic identification of *Paenibacillus* sp. TRMB57781.** (**A**) Colony morphology of TRMB57781 on LB agar medium. (**B**) Morphology of bacterial cells and spores of TRMB57781 under scanning electron microscope (1000× magnification). (**C**) Phylogenetic tree of TRMB57781, 24 closely related type strains of the genus *Paenibacillus*, and the outgroup (*E. coli* K-12 MG1655^T^) based on 16S rRNA gene sequences (constructed using the maximum likelihood method in MEGA X software with 1000 bootstrap replications; values at nodes represent confidence levels; scale bar indicates 0.02 nucleotide substitution rate).

Colonies of strain TRMB57781 on LB agar were circular, milky white, and viscous, with cells appearing as uniform rods (2.0–3.5 × 0.6–0.8 µm) under SEM (Figure 1A,B). Phylogenetic analysis based on 16S rRNA gene sequences placed the strain in a cluster with *Paenibacillus peoriae* (bootstrap = 72%; sequence similarity = 99.44%), yet it formed a distinct sublineage, warranting its provisional assignment to the genus *Paenibacillus* (*Paenibacillus* sp.). Phenotypic comparison with the three most closely related type strains revealed clear differences in the utilization of D-mannose, L-fucose, potassium gluconate, and sorbitol (Table 1), providing supplementary evidence for its taxonomic distinctiveness.

### 3.2. Genomic Analysis of TRMB57781 and Antimicrobial Potential of Strain TRMB57781

**Figure 2:**
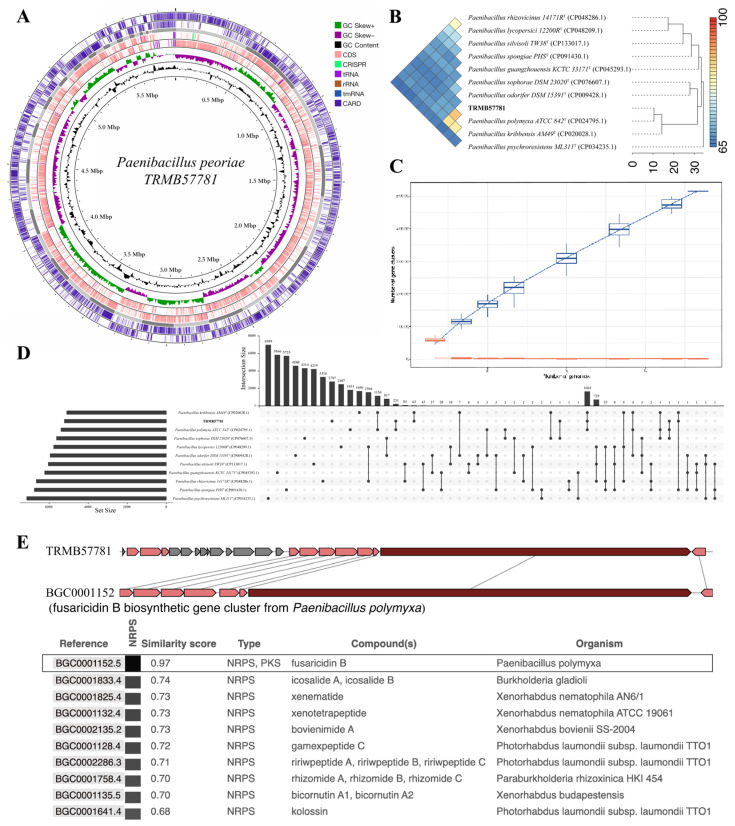
Genomic characteristics and comparative genomic analysis of TRMB57781. (**A**) Circular genome map of *Paenibacillus* strain TRMB57781. (**B**) Genome similarity analysis. (**C**) Pan-genome and core-genome rarefaction curves. (**D**) Upset plot of gene cluster intersections among *Paenibacillus* strains. (**E**) Comparison of secondary metabolite biosynthetic gene clusters (BGCs).

The whole-genome next-generation sequencing revealed 606 contigs in the assembled genome, totaling 5,900,343 bp. Bakta identified 5396 genomic features (Figure 2A), comprising 5209 CDS, 116 *tRNA*, 8 *rRNA*, and 50 ncRNA regions. Utilizing the CARD database, RGI in Proksee predicted 4788 antibiotic resistance-related features, encompassing resistance genes and mutation sites.

Whole-genome comparative analysis was performed using the IPGA pipeline. In the ANI clustering analysis (Figure 2B), strain TRMB57781 clustered most closely with *Paenibacillus polymyxa* ATCC 842^T^ and *Paenibacillus kribbensis* AM49^T^; however, the pairwise FastANI values between TRMB57781 and these two strains were only 89.53% and 86.69%, respectively, both falling well below the widely accepted species threshold of 95–96%. The ANI values with the remaining seven reference strains were all below 70% (Table S1), and the heatmap displayed predominantly deep blue coloration, indicating low genome-wide similarity and a distant genetic relationship. These results demonstrate that strain TRMB57781 cannot be assigned to any of the known species included in this comparison. Pan-genome and core genome rarefaction curves (Figure 2C) further illustrated the genomic diversity and conservation within the analyzed group, quantifying the “conserved-variable” gene components. Collectively, these findings suggest that the species in this genus maintain essential survival mechanisms through a “conserved core + variable pan-genome” model, adapt to diverse environments via genetic diversity, and retain substantial untapped genetic potential.

The Upset plot (Figure 2D) from gene cluster intersection analysis systematically illustrated gene cluster sharing patterns among 11 *Paenibacillus* strains. It facilitated precise identification of three genetic unit types: core gene clusters shared by all strains (representing the species-conserved functional modules), unique gene clusters exclusive to individual strains (primarily linked to niche specialization), and strain groups with close gene sharing. These findings validate the “conserved core-diversified pan-genome” genomic architecture of the genus *Paenibacillus* at the gene cluster level, offering direct insights into the genetic underpinnings of niche differentiation and functional innovation.

Using antiSMASH (Figure 2E) to predict secondary metabolite biosynthetic gene clusters and aligning them with the MIBiG database revealed that the fusaricidin B biosynthetic gene cluster in strain TRMB57781 shared 97% similarity with the homologous cluster in *Paenibacillus polymyxa.* The highly conserved modular structure suggests the strain has the potential to produce fusaricidin B through its metabolic pathways.

### 3.3. Assessment of the Antimicrobial Efficacy of TRMB57781 and Enhancement of the Optimal Fermentation Medium

**Figure 3:**
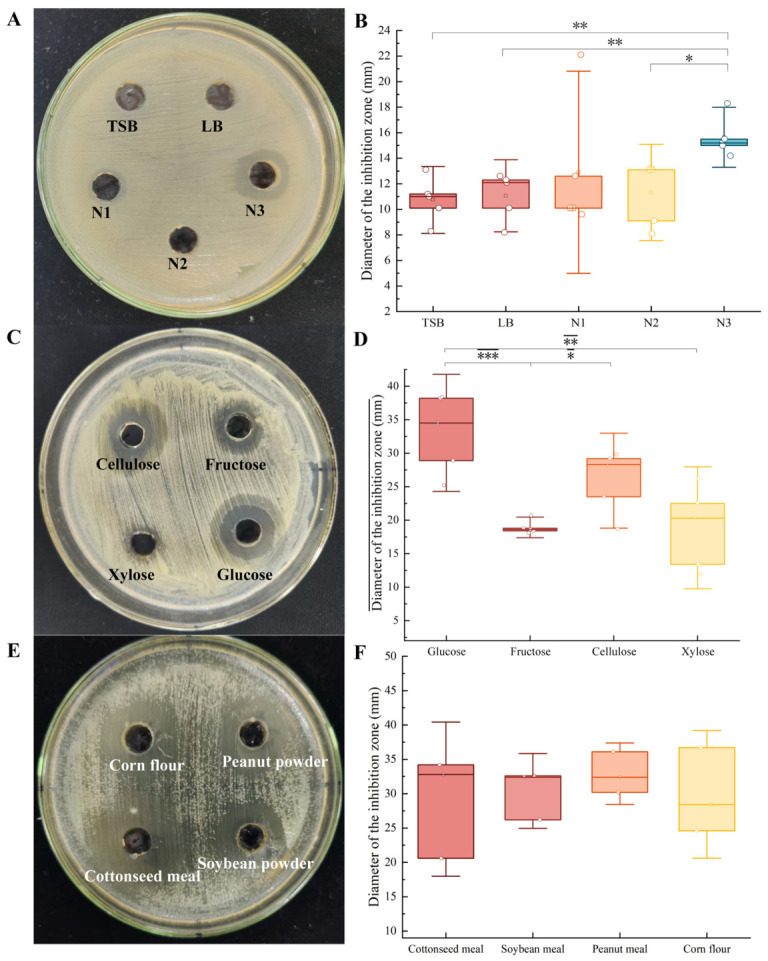
Evaluation of antimicrobial activity of TRMB57781 fermentation broth against *E. amylovora* and optimization of the optimal fermentation medium. (**A**,**B**) Screening of basal culture media for TRMB57781: Agar well diffusion (Oxford cup) assay plates and box plots of inhibition zone diameters. Asterisks indicate significant differences between the indicated groups (Student’s *t*-test, *p* < 0.05). (**C**,**D**) Screening of optimal carbon sources for TRMB57781: Agar well diffusion (Oxford cup) assay plates and box plots of inhibition zone diameters. Asterisks indicate significant differences between the indicated groups (Student’s *t*-test, *p* < 0.05). (**E**,**F**) Screening of optimal nitrogen sources for TRMB57781: Agar well diffusion (Oxford cup) assay plates and box plots of inhibition zone diameters. Asterisks indicate significant differences between the indicated groups (Student’s *t*-test): * *p* < 0.05; ** *p* < 0.01; *** *p* < 0.001.

The antibacterial activity of strain TRMB57781 against *E. amylovora* was compared across five media. Among them, N3 medium produced the largest inhibition zone (15.64 mm, Figure 3A) and was therefore selected as the basal medium for further optimization.

Based on N3, four carbon sources were evaluated. Glucose yielded the largest inhibition zone (13.56 mm), followed by fructose (13.26 mm), while xylose and cellulose gave markedly smaller zones (9.66 mm and 9.86 mm, respectively; Figure 3B). Glucose was therefore identified as the optimal carbon source.

Using glucose as the fixed carbon source, four nitrogen sources were then compared. Cottonseed meal produced the largest inhibition zone (12.20 mm), surpassing soybean meal (10.12 mm), peanut meal (9.24 mm), and corn steep liquor powder (8.64 mm). Accordingly, cottonseed meal was established as the optimal nitrogen source.

### 3.4. Evaluation of the Biocontrol Effects of TRMB57781 Fermentation Broth on Various Components of In Vitro Fragrant Pear Materials

**Figure 4:**
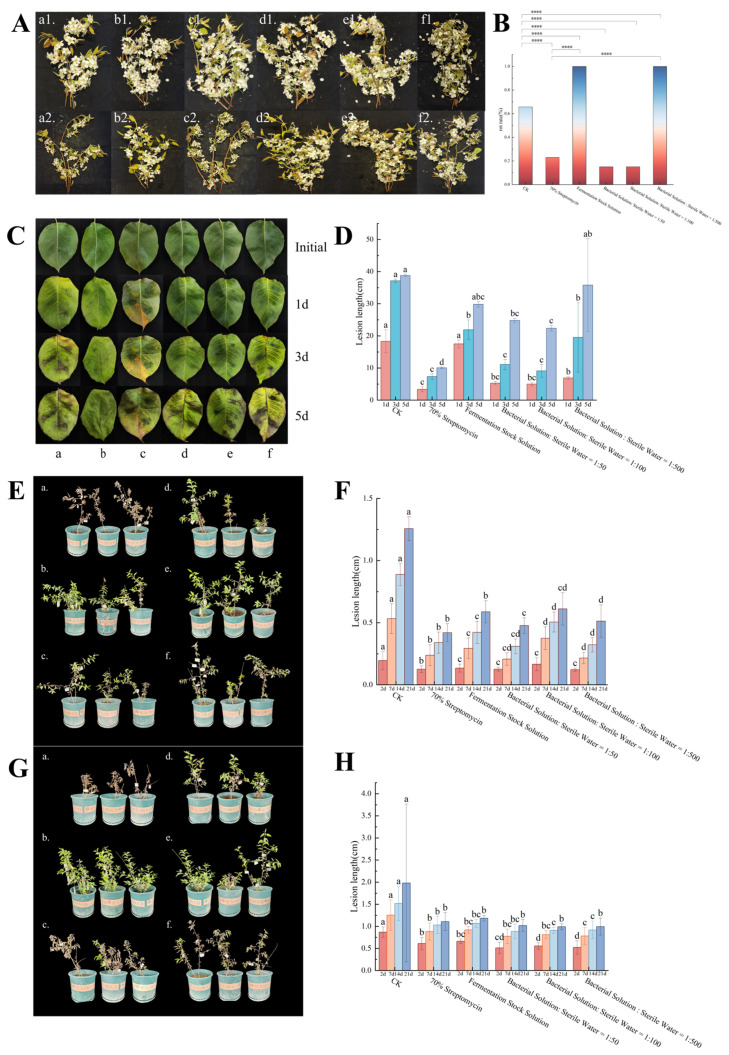
Multi-system evaluation of the control effect of strain TRMB57781 against pear fire blight. (**A**,**B**) show the infection phenotypes on the 3rd day of the in vitro inflorescence experiment, and the dual-axis bar and line chart illustrate the statistics of rotten inflorescence numbers among different treatment groups. The left vertical axis of the chart represents the total number of inflorescences, while the right vertical axis denotes the rot rate (%). Purple bar graphs indicate the rot rate, calculated as (number of rotten inflorescences/total number of inflorescences) × 100%. Treatments are as follows: a: fresh sterile N3S culture medium blank control; b: 70% agricultural streptomycin sulfate soluble powder positive control; c: undiluted fermentation broth of TRMB57781; d: 50-fold diluted fermentation broth; e: 100-fold diluted fermentation broth; f: 500-fold diluted fermentation broth. Different lowercase letters indicate significant differences between groups (chi-square test, *p* < 0.05). Asterisks indicate significant differences compared to the control: **** *p* < 0.0001. Numbers 1 and 2 in the subfigure labels (e.g., a1, a2) indicate the treatment stage: 1 = pre-treatment (spray), 2 = post-inoculation (pathogen challenge). (**C**,**D**) present the phenotypes from the in vitro leaf activity assay of TRMB57781 fermentation broth and combined bar charts of the statistical analysis of rotten leaf numbers among different treatment groups (unit: mm). Treatments are consistent with those in (**B**) (corresponding to a–f). Leaf phenotype changes were recorded at 0 d, 1 d, 3 d, and 5 d, along with the quantitative results of leaf lesion length under different treatments (mean ± SD). Asterisks indicate significant differences between the indicated groups (Student’s *t*-test, *p* < 0.05). (**E**,**F**) display the infection phenotypes of Kuerle fragrant pear seedlings on the 21st day of the fire blight prevention experiment and the statistical analysis of lesion length in each treatment group (unit: mm). Treatments are consistent with those in (**B**) (corresponding to a–f). Different lowercase letters indicate significant differences between groups (one-way ANOVA followed by Tukey’s HSD test, *p* < 0.05). (**G**,**H**) show the infection phenotypes of Kuerle fragrant pear seedlings on the 21st day of the fire blight treatment experiment and the statistical analysis of lesion length in each treatment group (unit: mm). Treatments are consistent with those in (**B**) (corresponding to a–f). Different lowercase letters indicate significant differences between groups (one-way ANOVA followed by Tukey’s HSD test, *p* < 0.05).

The results of the TRMB57781 fermentation broth activity determination on in vitro inflorescences (Figure 4A,B) revealed that both the control group and the undiluted bacterial solution treatment group displayed typical flower rot symptoms in the nectaries, anthers, and calyces of inflorescences, indicating that the undiluted bacterial solution did not mitigate the infection. However, the incidence of diseased inflorescences in the fermentation broth treatment groups, particularly in the 1:50 and 1:100 dilution groups, was significantly lower than that in the control group. The control efficiency index of the 1:50 dilution group reached 84.62%, surpassing that of agricultural streptomycin at 69.48% by 15.14%.

The in vitro leaf activity assessment revealed a declining trend in the control efficacy of the bacterial solution over time, possibly attributed to volatilization. While agricultural streptomycin exhibited superior overall control efficiency, the 1:100 diluted bacterial solution demonstrated control efficiencies of 72.84% on day 1 and 75.49% on day 3, closely approaching those of streptomycin (81.58% and 80.23%). This dilution emerged as the optimal choice, balancing antimicrobial effectiveness and plant safety. Despite streptomycin’s short-term high efficacy, ongoing monitoring of its potential side effects is warranted.

In the potted plant experiment, the preventive treatment (Figure 4E,F) demonstrated a significant reduction in lesion length of potted plants infected with *E. amylovora* when sprayed with fermentation broth of varying concentrations compared to the blank control group. This effect was comparable to that of agricultural streptomycin, indicating a strong biological preventive efficacy. In the therapeutic treatment (Figure 4G,H), spraying fermentation broth post pathogen inoculation notably improved lesion length to a degree equivalent to agricultural streptomycin, showcasing its remarkable biological therapeutic potential.

### 3.5. Extraction of Secondary Metabolites from TRMB57781 and Initial Investigation of Antimicrobial Agents

**Figure 5:**
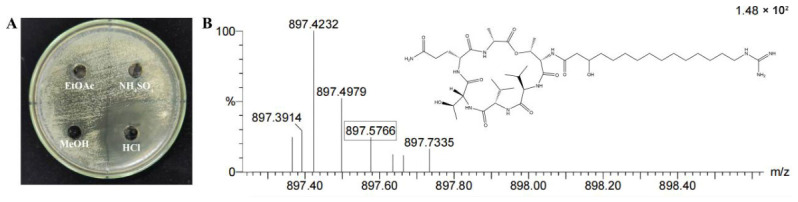
Identification of active components in TRMB57781 fermentation products. (**A**) Antimicrobial activity of crude extracts from TRMB57781 fermentation products against pear fire blight pathogen. The antimicrobial activity of secondary metabolites from different treatments was determined using the Oxford cup method (EtOAc: low-polarity or non-polar crude extract; NH_4_SO_4_: crude protein extract; MeOH: medium-polarity crude extract; HCl: crude lipopeptide extract). (**B**) Time-of-flight mass spectrometry (TOF MS) diagram of crude lipopeptide extract using ultra-performance liquid chromatography-quadrupole time-of-flight mass spectrometry in electrospray positive ion mode (ES+).

A “targeted extraction, precise identification, and genetic corroboration” strategy was adopted to identify the antimicrobial agent of strain TRMB57781. The crude lipopeptide fraction was obtained from the fermentation broth by HCl precipitation (Figure 5A), and subsequently analyzed by high-resolution time-of-flight mass spectrometry (HR-TOF MS, ES^+^ mode). Among 130 candidate molecular formulas, one matched the theoretical characteristics of fusaricidin B. The measured mass-to-charge ratio (*m*/*z*) of 897.5766 deviated from the theoretical mass of 897.5773 by −0.7 mDa (−0.8 ppm), with a deduced degree of unsaturation of 9.5, consistent with its polycyclic structure (Figure 5B). These results strongly support the identification of the compound as fusaricidin B.

Whole-genome analysis and antiSMASH verification confirmed the presence of the fusaricidin B biosynthetic gene cluster in the strain, annotated as a Polyketide + NRP-type lipopeptide, exhibiting 100% similarity with the reference sequence and indicating the genetic capacity to produce this compound.

Taken together, both chemical detection and genetic evidence strongly support the production of fusaricidin B by strain TRMB57781. Notably, antimicrobial activity was exclusively associated with the HCl-precipitated crude lipopeptide fraction. The high-resolution mass spectrometric and genomic data are highly consistent with the structural and biosynthetic expectations for fusaricidin B, suggesting its presence in this active fraction. This indicates that fusaricidin B is the key active component responsible for the antagonism against *E. amylovora*. Although the possible presence of other minor metabolites in the crude fraction cannot be entirely excluded, the current data identify fusaricidin B as the primary antimicrobial agent in this fraction.

## 4. Discussion

One of the core demands in the current development of green agriculture is the exploration of microbial resources with both ecological safety and high-efficiency functionality to replace traditional chemical pesticides and fertilizers, thereby reducing environmental loads and ensuring agricultural product safety. Among numerous candidate microorganisms, the genus *Paenibacillus*, endowed with remarkable metabolic diversity and enormous potential for secondary metabolite synthesis, has emerged as a research frontier in the fields of agricultural biological control and plant growth regulation. A large body of cutting-edge studies has confirmed that *Paenibacillus* strains can not only improve soil fertility through nutrient activation processes such as phosphorus solubilization, potassium solubilization, and nitrogen fixation, but also synthesize substances including siderophores and plant hormones to directly promote plant growth [40,41,42,43]; more importantly, the secondary metabolites produced by them, such as antibiotics, antimicrobial peptides, and volatile organic compounds, can precisely target and inhibit a variety of plant pathogens, exhibiting irreplaceable advantages in the green control of plant diseases [44,45,46]. *Paenibacillus* has become a key research focus for researchers worldwide in addressing intractable bacterial diseases.

Fire blight, a devastating disease caused by *E. amylovora*, has been listed as an important global plant quarantine pest. Its host range covers *Pyrus* species and various Rosaceae plants, inflicting substantial economic losses on the global fruit tree industry. In the main producing areas of Kuerle fragrant pear in southern Xinjiang, China, the outbreak and spread of fire blight in recent years have not only led to a sharp decline in pear yield, but also severely impacted the stability of the regional agricultural economy and farmers’ incomes. Currently, the control of fire blight still relies on chemical agents; however, problems arising from chemical control, such as environmental residues and increased pathogen resistance, have become bottlenecks restricting the sustainable development of the industry. Therefore, the exploration of efficient and safe biological control microbial resources and the construction of green control technology systems have become frontier research hotspots in the field of fire blight control. Owing to the aforementioned unique advantages, *Paenibacillus* strains have naturally become key objects for exploration and utilization in this field.

Notably, extreme habitats (e.g., industrial wastewater, saline-alkali land, high-temperature environments) often harbor microbial resources with special metabolic mechanisms and excellent functional potential due to their unique physical and chemical conditions. The exploration and utilization of such resources have become a frontier trend in the fields of microbial ecology and agricultural biotechnology [47,48]. Cutting-edge studies have demonstrated that *Paenibacillus* strains derived from extreme habitats typically contain more unique secondary metabolite synthesis gene clusters in their genomes. This is closely associated with their evolutionary strategies for adapting to extreme environments and endows them with inherent advantages as efficient biological control strains [47,48]. Against this cutting-edge background, *Paenibacillus* strain TRMB57781, isolated from the special extreme habitat of industrial wastewater from Alar Chemical Plant in this study, exhibits antimicrobial activity against *E. amylovora*. This finding precisely confirms the frontier cognition that extreme habitats serve as reservoirs of efficient biological control resources, providing new directions and ideas for the exploration of biological control strains against fire blight. Comparative genomic analyses further revealed that strain TRMB57781 exhibited pairwise FastANI values of 89.53% and 86.69% with its closest relatives, *Paenibacillus polymyxa* ATCC 842^T^ and *P. kribbensis* AM49^T^, respectively, both well below the widely accepted species demarcation threshold of 95–96%. Pan-genome and core genome rarefaction curves demonstrated a “conserved core + variable pan-genome” architecture, suggesting that this strain retains essential metabolic functions while harboring substantial genetic diversity that may contribute to its ecological adaptability and biocontrol potential.

In frontier research on the biological control mechanisms of *Paenibacillus*, lipopeptide compounds are regarded as one of the core functional substances due to their broad-spectrum antimicrobial activity and environmentally friendly characteristics [48]. Among them, fusaricidin family lipopeptides have become the focus of research on *Paenibacillus*-mediated control of bacterial diseases, attributed to their high-efficiency inhibitory effects on Gram-negative bacteria [49]. Cutting-edge studies have confirmed that fusaricidin family compounds exert antimicrobial effects by disrupting the integrity of pathogen cell membranes, and the conservation of their synthesis gene clusters is closely correlated with the biological control efficacy of the strains [49]. This study identified fusaricidin B as the core antimicrobial active component of TRMB57781. In the genome of strain TRMB57781, antiSMASH predicted a fusaricidin B biosynthetic gene cluster spanning approximately 45.4 kb (14,224–59,641 nt). The core NRPS gene (ctg27_32) encodes a multi-modular enzyme of 7907 amino acids. This NRPS assembly line comprises six modules, each containing a condensation domain (C) and an adenylation domain (A), with the domain organization arranged as C-A, C-A-E, C-A, C-A-E, C-A-E, and C-A-TE. The presence of epimerization (E) domains in modules 2, 4, and 5 is consistent with the D-configuration of the corresponding amino acid residues in fusaricidin B (D-Val, D-allo-Thr, D-Asn), while the terminal thioesterase (TE) domain mediates chain release and macrocyclization. A-domain substrate predictions for Thr, D-Val, D-Thr, and D-Asn matched the known amino acid sequence of fusaricidin B, whereas the substrates for modules 3 and 6 were returned as unknown due to insufficient database matches—a common occurrence in nonribosomal peptide prediction. The overall modular architecture is highly conserved compared with well-characterized fusaricidin B producers such as *P. polymyxa* E681 and *P. polymyxa* SC2, with only minor variations in substrate specificity. The gene cluster shares 97% sequence similarity with the annotated fusaricidin B cluster in the MIBiG database, providing strong genetic evidence for the capacity of this strain to produce fusaricidin B. This result is not an isolated case but further corroborates the core conclusion from frontier research that “fusaricidin family compounds are the key material basis for *Paenibacillus* to control bacterial diseases”. Meanwhile, it enriches the application scenarios of fusaricidin family compounds in fire blight control and provides a new strain for the industrial application of such compounds. It should be noted that the identification of fusaricidin B in this study was based on high-resolution mass spectrometry (mass error of −0.8 ppm) and genome mining evidence. More definitive structural confirmation, such as MS/MS fragmentation matching or co-injection with an authentic reference standard, will be pursued in future studies to provide orthogonal validation.

From the perspective of the global frontier in green agriculture development, the practical application of biological control microorganisms depends not only on their efficiency but also on their ecological adaptability and environmental safety [48]. Cutting-edge studies have pointed out that microbial strains derived from natural or special habitats, due to their long-term adaptation to natural ecosystems, are more likely to colonize and exert stable effects in agricultural ecosystems without disrupting the soil microecological balance [50,51]. *Paenibacillus* strains have become hotspots in the field of biological control precisely because they inherently possess such excellent ecological adaptability. Moreover, strains from extreme habitats show broader application prospects due to their unique environmental adaptation capabilities [47]. The exploration and verification of the relevant strain in this study are precisely in line with this frontier development direction, providing a typical case for the development and utilization of microbial resources from extreme habitats.

In summary, under the background of global agricultural green transformation, the exploration and utilization of efficient biological control microbial resources is an inevitable trend. As a core object of frontier research, *Paenibacillus*, with the resource value of its extreme habitat-derived strains and the functional potential of fusaricidin family compounds, provides a new path for the green control of intractable diseases such as fire blight. The exploration in this study is merely a supplement and confirmation to this frontier field. Future research should focus on cutting-edge fermentation engineering and synthetic biology technologies to functionally validate the fusaricidin B biosynthetic pathway, optimize formulation strategies for field applications, further tap into the potential of microbial resources from extreme habitats, promote the industrial transformation of biological control strains, and contribute to the high-quality development of green agriculture.

## 5. Conclusions

In this study, a *Paenibacillus peoriae* strain, TRMB57781, with potent antagonistic activity against *Erwinia amylovora* was isolated from chemical plant wastewater and characterized through polyphasic taxonomy. Comparative genomic analyses revealed a “conserved core + variable pan-genome” architecture, with ANI values falling below the species demarcation threshold, underscoring the strain’s unique taxonomic position and genetic background. Genome mining combined with chemical identification confirmed fusaricidin B as the principal antimicrobial metabolite, and its biosynthetic gene cluster was found to be highly conserved. In vitro and in planta assays demonstrated that TRMB57781 fermentation broth provided significant preventive and therapeutic efficacy against pear fire blight, with control effects at optimized dilutions comparable to or exceeding those of agricultural streptomycin. Collectively, this work not only delivers a promising biocontrol candidate for the management of pear fire blight, but also establishes an integrated genomic-to-functional framework linking biosynthetic potential to antibacterial output. More definitive structural validation, such as MS/MS fragmentation matching or co-injection with an authentic standard, should be pursued in future investigations.

## Figures and Tables

**Table 1:** Differential physiological and biochemical characteristics between strain TRMB57781 and phylogenetically related species of the genus *Paenibacillus*.

Test Items	Result			
	TRMB57781	*P. peoriae* BD-57^T^	*P. polymyxa* DSM 36^T^	*P. ottowii* MS2379^T^
**Carbohydrate Acidogenesis**				
D-fructose	+	+	+	+
D-fucose	−	−	−	−
D-galactose	+	+	+	+
D-mannose	−	+	+	+
D-ribose	+	+	+	Ne
D-tagatose	−	−	−	−
erythritol	−	−	−	−
L-arabinose	+	+	+	+
L-fucose	+	−	−	−
L-sorbose	−	−	−	−
L-xylose	−	−	−	−
myo-inositol	+	Ne	−	−
Potassium 2-ketogluconate	−	−	−	Ne
Potassium 5-ketogluconate	−	−	−	Ne
potassium gluconate	+	Ne	Ne	−
salicin	+	+	+	+
sorbitol	+	Ne	Ne	−
xylitol	−	−	−	−
Gentiobiose	+	+	+	+
Maltose	+	+	+	+
Raffinose	+	+	+	+
**Enzyme Activity**				
alpha-galactosidase	+	Ne	+	Ne
beta-galactosidase	+	Ne	+	Ne

Taxa: +, Positive; −, negative; Ne, no data available. Data obtained from BacDive (https://doi.org/10.1093/nar/gkab961).

## Data Availability

The whole-genome sequence data will be submitted to NCBI GenBank, and the accession number will be provided during the review process. Dataset available on request from the authors.

## Supplementary Materials

The following supporting information can be downloaded at: https://www.mdpi.com/article/10.3390/biology15100764/s1, Table S1. Average nucleotide identity (ANI) values between strain TRMB57781 and closely related Paenibacillus type strains.

## Abbreviations

ANI	Average Nucleotide Identity
BGCs	Biosynthetic Gene Clusters
CARD	Comprehensive Antibiotic Research Database
CDS	Coding Sequence
CFU	colony-forming unit
CTAB	cetyltrimethylammonium bromide
DNA	Deoxyribonucleic acid
*E. amylovora*	*Erwinia amylovora*
ESI	electrospray ionization
ES^+^	electrospray ionization positive mode
H_2_O_2_	hydrogen peroxide
HR-TOF MS	High-Resolution Time-of-Flight Mass Spectrometry
LB	Lysogeny Broth
m/z	mass-to-charge ratio
NGS	next-generation sequencing
ncRNA	non-coding RNA
OD_600_	Optical Density at 600 nm
RPM	revolutions per minute
rRNA	ribosomal RNA
SEM	scanning electron microscopy
SPSS	Statistical Package for the Social Sciences
TSB	Tryptic Soy Broth
tRNA	transfer RNA
UPLC-Q-TOF MS	Ultra-Performance Liquid Chromatography-Quadrupole Time-of-Flight Mass Spectrometry

## References

[1] Myung I.S., Lee J.Y., Yun M.J., Lee Y.H., Lee Y.K., Park D.H., Oh C.S. (2016). Fire Blight of Apple, Caused by *Erwinia amylovora*, a New Disease in Korea. Plant Dis..

[2] Zhao Y.Q., Tian Y.L., Wang L.M., Geng G.M., Zhao W.J., Hu B.S., Zhao Y.F. (2019). Fire blight disease, a fast-approaching threat to apple and pear production in China. J. Integr. Agric..

[3] Vrancken K., Holtappels M., Schoofs H., Deckers T., Valcke R. (2013). Pathogenicity and infection strategies of the fire blight pathogen *Erwinia amylovora* in Rosaceae: State of the art. Microbiology.

[4] Chen X., Zhang M., Sun M., Liu Y., Li S., Song B., Li M., Zhang S., Wang R., Li J. (2022). Genome-wide genetic diversity and IBD analysis reveals historic dissemination routes of pear in China. Tree Genet. Genomes.

[5] Chen C., Sun L., Sheng Q., Han J., Fu B., Luo M. (2024). Influences of Interactions Between *Erwinia amylovora* and *Valsa pyri* on Growth and Pathogenicity. Acta Hortic. Sin..

[6] Shen T., Ye M., Xu Y., Ding B., Li H., Zhang L., Wang J., Tian Y., Hu B., Zhao Y. (2024). *Cytospora pyri* promotes *Erwinia amylovora* virulence by providing metabolites and hyphae. J. Integr. Agric..

[7] Slack S.M., Schachterle J.K., Sweeney E.M., Kharadi R.R., Peng J., Botti-Marino M., Bardaji L., Pochubay E.A., Sundin G.W. (2022). In-Orchard Population Dynamics of *Erwinia amylovora* on Apple Flower Stigmas. Phytopathology.

[8] Kharadi R.R., Schachterle J.K., Yuan X., Castiblanco L.F., Peng J., Slack S.M., Zeng Q., Sundin G.W. (2021). Genetic Dissection of the *Erwinia amylovora* Disease Cycle. Annu. Rev. Phytopathol..

[9] Santander R.D., Català-Senent J.F., Figàs-Segura À., Biosca E.G. (2020). From the roots to the stem: Unveiling pear root colonization and infection pathways by *Erwinia amylovora*. FEMS Microbiol. Ecol..

[10] Tancos K.A., Cox K.D. (2017). Effects of Consecutive Streptomycin and Kasugamycin Applications on Epiphytic Bacteria in the Apple Phyllosphere. Plant Dis..

[11] McGhee G.C., Guasco J., Bellomo L.M., Blumer-Schuette S.E., Shane W.W., Irish-Brown A., Sundin G.W. (2011). Genetic Analysis of Streptomycin-Resistant (Sm^R^) Strains of *Erwinia amylovora* Suggests that Dissemination of Two Genotypes Is Responsible for the Current Distribution of Sm^R^
*E. amylovora* in Michigan. Phytopathology.

[12] Borba M.C., Meredith C.L., Dhar B.C., Aćimović S.G. (2023). Proof of concept for management of shoot blight and fire blight cankers on pear with preventive spray applications of giant knotweed extract. Front. Hortic..

[13] Mendes R.J., Regalado L., Rezzonico F., Tavares F., Santos C. (2024). Deciphering Fire Blight: From *Erwinia amylovora* Ecology to Genomics and Sustainable Control. Horticulturae.

[14] Yi A., Chen L., Wei J., Zhang Z.-S., Li W., Shi Y., Wang B., Wang X., Cui Z.-N. (2025). Synthesis and Biological Evaluation of Disulfides Based on Garlic Extract as Type III Secretion System Inhibitors against *Erwinia amylovora*. J. Agric. Food Chem..

[15] Mahida D.K., Patel A., Sankhla M.S., Chavda B., Makwana V.M., Kumar K., Rawal R. (2025). Assessing the Ecotoxicological Impact of Copper-Based Nano-Pesticides: A Comprehensive Review. Biointerface Res. Appl. Chem..

[16] Manyi-Loh C., Mamphweli S., Meyer E., Okoh A. (2018). Antibiotic Use in Agriculture and Its Consequential Resistance in Environmental Sources: Potential Public Health Implications. Molecules.

[17] Luo L., Zhao C., Wang E., Raza A., Yin C. (2022). *Bacillus amyloliquefaciens* as an excellent agent for biofertilizer and biocontrol in agriculture: An overview for its mechanisms. Microbiol. Res..

[18] Xue Y., Zhang Y., Huang K., Wang X., Xing M., Xu Q., Guo Y. (2023). A novel biocontrol agent *Bacillus velezensis* K01 for management of gray mold caused by *Botrytis cinerea*. AMB Express.

[19] Cui W., He P., Munir S., He P., Li X., Li Y., Wu J., Wu Y., Yang L., He P. (2019). Efficacy of plant growth promoting bacteria *Bacillus amyloliquefaciens* B9601-Y2 for biocontrol of southern corn leaf blight. Biol. Control.

[20] Cui W., He P. (2022). Genome Sequence Resource of *Bacillus velezensis* Strain HC-8, a Native Bacterial Endophyte with Biocontrol Potential Against the Honeysuckle Powdery Mildew Causative Pathogen *Erysiphe lonicerae* var. *lonicerae*. Mol. Plant-Microbe Interact. MPMI.

[21] Dobrzyński J., Jakubowska Z., Kulkova I., Kowalczyk P., Kramkowski K. (2023). Biocontrol of fungal phytopathogens by *Bacillus pumilus*. Front. Microbiol..

[22] Alfiky A., L’HAridon F., Abou-Mansour E., Weisskopf L. (2022). Disease Inhibiting Effect of Strain *Bacillus subtilis* EG21 and Its Metabolites Against Potato Pathogens *Phytophthora infestans* and *Rhizoctonia solani*. Phytopathology.

[23] Wu Y., Zhou J., Li C., Ma Y. (2019). Antifungal and plant growth promotion activity of volatile organic compounds produced by *Bacillus amyloliquefaciens*. Microbiol. Open.

[24] Zhang M., Li X., Pan Y., Qi D., Zhou D., Chen Y., Feng J., Wei Y., Zhao Y., Li K. (2024). Biocontrol mechanism of *Bacillus siamensis* sp. QN_2_MO-1 against tomato fusarium wilt disease during fruit postharvest and planting. Microbiol. Res..

[25] Zhang Y., Yang Y., Zhang L., Zhang J., Zhou Z., Yang J., Hu Y., Gao X., Chen R., Huang Z. (2023). Antifungal mechanisms of the antagonistic bacterium *Bacillus mojavensis* UTF-33 and its potential as a new biopesticide. Front. Microbiol..

[26] Zhang H., Liu Y., Wu G., Dong X., Xiong Q., Chen L., Xu Z., Feng H., Zhang R. (2021). *Bacillus velezensis* tolerance to the induced oxidative stress in root colonization contributed by the two-component regulatory system sensor ResE. Plant Cell Environ..

[27] Yang J., Li S., Zhou X., Du C., Fang J., Li X., Zhao J., Ding F., Wang Y., Zhang Q. (2025). *Bacillus amyloliquefaciens* promotes cluster root formation of white lupin under low phosphorus by mediating auxin levels. Plant Physiol..

[28] Shao D., He Y., Zhai Y., Yang X., Guo Z., Tan J., Wei M. (2025). Mechanisms of tomato growth promotion in three soils after applying *Bacillus* combinations. Soil Tillage Res..

[29] Xie S., Si H., Xue Y., Zhou R., Wang S., Duan Y., Niu J., Wang Z. (2024). Efficacy of rhizobacteria *Paenibacillus polymyxa* SY42 for the biological control of *Atractylodes chinensis* root rot. Microb. Pathog..

[30] Li X., Wang J., Lv Y., Zhao L., Jiang W., Lv J., Xu X., Yu Y., Liu Y., Chen X. (2025). Screening and identification of *Paenibacillus polymyxa* GRY-11 and its biological control potential against apple replant disease. Folia Microbiol..

[31] Shi Q., Zhang J., Fu Q., Hao G., Liang C., Duan F., Zhao H., Song W. (2024). Biocontrol efficacy and induced resistance of *Paenibacillus polymyxa* J2-4 against *Meloidogyne incognita* infection in cucumber. Phytopathology.

[32] Mahmoud M., Seguin P., Faucher S.P., Jabaji S. (2026). Characterization and antifungal properties against *Botrytis cinerea* of bacteria isolated from hemp seed oil. Can. J. Microbiol..

[33] Li E., Liu K., Yang S., Li L., Ran K., Sun X., Qu J., Zhao L., Xin Y., Zhu F. (2024). Analysis of the complete genome sequence of *paenibacillus* sp. lzh-N1 reveals its antagonistic ability. BMC Genom..

[34] Du J., Li H., Liu S., Wu L., Liu Y., Li Y., Zhao X., Gao Q., Dong J., Lei C. (2025). Hyaluronic acid-functionalized nanoarmor enhances the stable colonization ability of *Paenibacillus polymyxa* JF_P68 and boosts its biological control efficacy against pear anthracnose. Pest Manag. Sci..

[35] Yu C., Yang X., Liang X., Song Y., Zhu L., Xing S., Yang Y., Gu Q., Borriss R., Dong S. (2023). Fusaricidin produced by the rhizobacterium *Paenibacillus polymyxa* NX20 is involved in the biocontrol of postharvest plant-pathogenic oomycete *Phytophthora capsici*. Postharvest Biol. Technol..

[36] Blin K., Shaw S., Vader L., Szenei J., Reitz Z.L., Augustijn H.E., Cediel-Becerra J.D.D., de Crécy-Lagard V., Koetsier R.A., Williams S.E. (2025). antiSMASH 8.0: Extended gene cluster detection capabilities and analyses of chemistry, enzymology, and regulation. Nucleic Acids Res..

[37] Liu D., Zhang Y., Fan G., Sun D., Zhang X., Yu Z., Wang J., Wu L., Shi W., Ma J. (2022). IPGA: A handy integrated prokaryotes genome and pan-genome analysis web service. iMeta.

[38] Prjibelski A., Antipov D., Meleshko D., Lapidus A., Korobeynikov A. (2020). Using SPAdes De Novo Assembler. Curr. Protoc. Bioinform..

[39] Grant J.R., Enns E., Marinier E., Mandal A., Herman E.K., Chen C.-Y., Graham M., Van Domselaar G., Stothard P. (2023). Proksee: In-depth characterization and visualization of bacterial genomes. Nucleic Acids Res..

[40] Roy M., Kang B., Yang S., Choi H., Choi K. (2024). Characterization of Tomato Seed Endophytic Bacteria as Growth Promoters and Potential Biocontrol Agents. Plant Pathol. J..

[41] Li Z., Lin Y., Song F., Zheng R., Huang Q. (2024). Isolation and characterization of *Paenibacillus peoriae* JC-3jx from *Dendrobium nobile*. BioTechniques.

[42] Devkota P., Kloepper J.W., Enebak S.A., Eckhardt L.G. (2020). Towards biocontrol of ophiostomatoid fungi by plant growth-promoting rhizobacteria. Biocontrol Sci. Technol..

[43] Liu K., Newman M., McInroy J.A., Hu C.-H., Kloepper J.W. (2017). Selection and Assessment of Plant Growth-Promoting Rhizobacteria for Biological Control of Multiple Plant Diseases. Phytopathology.

[44] Yadav D.R., Adhikari M., Kim S.W., Kim H.S., Lee Y.S. (2021). Suppression of Fusarium Wilt Caused by *Fusarium oxysporum* f. sp. *lactucae* and Growth Promotion on Lettuce Using Bacterial Isolates. J. Microbiol. Biotechnol..

[45] Jiang A., Zou C., Xu X., Ke Z., Hou J., Jiang G., Fan C., Gong J., Wei J. (2022). Complete genome sequence of biocontrol strain *Paenibacillus peoriae* HJ-2 and further analysis of its biocontrol mechanism. BMC Genom..

[46] Dunlap C.A., Lueschow S., Carrillo D., Rooney A.P. (2017). Screening of bacteria for antagonistic activity against phytopathogens of avocados. Plant Gene.

[47] Grady E.N., MacDonald J., Liu L., Richman A., Yuan Z.-C. (2016). Current knowledge and perspectives of *Paenibacillus*: A review. Microb. Cell Fact..

[48] Lal S., Tabacchioni S. (2009). Ecology and biotechnological potential of *Paenibacillus polymyxa*: A minireview. Indian J. Microbiol..

[49] Yu C., Chen H., Zhu L., Song Y., Jiang Q., Zhang Y., Ali Q., Gu Q., Gao X., Borriss R. (2023). Profiling of Antimicrobial Metabolites Synthesized by the Endophytic and Genetically Amenable Biocontrol Strain *Bacillus velezensis* DMW1. Microbiol. Spectr..

[50] Tiwari S., Prasad V., Lata C. (2019). *Bacillus*: Plant Growth Promoting Bacteria for Sustainable Agriculture and Environment. New and Future Developments in Microbial Biotechnology and Bioengineering.

[51] Seldin L., Logan N.A., Vos P. (2011). *Paenibacillus*, Nitrogen Fixation and Soil Fertility. Endospore-Forming Soil Bacteria.

