# Supplementary material for: Antagonistic Activity and Molecular Insights into the Biocontrol of Korla Pear Fire Blight by Paenibacillus sp. TRMB57781
Source: Biology (Basel). 2026 May 11;15(10):764. doi: 10.3390/biology15100764 (PMC13203398; doi:10.3390/biology15100764)
Supplement: Supplementary file 1 [file biology-15-00764-s001.zip › biology-4277723-supplementary.pdf]

Table S1. Average nucleotide identity (ANI) values between strain TRMB57781 and closely related Paenibacillus type strains.

Reference strain	Strain designation	ANI (%)
Paenibacillus polymyxa	ATCC 842^T	89.53
Paenibacillus kribbensis	AM49^T	86.69
Paenibacillus odorifer	DSM 15391^T	68.79
Paenibacillus sophorae	DSM 23020^T	68.6
Paenibacillus guangzhouensis	KCTC 33171^T	67.45
Paenibacillus rhizovicinus	14171R	66.98
Paenibacillus spongiae	PHS	66.91
Paenibacillus lycopersici	12200R	66.82
Paenibacillus silvisoli	TW38^T	66.72
Paenibacillus psychroresistens	ML311^T	65.63

Note: Pairwise FastANI values were calculated using the IPGA pipeline. The widely accepted ANI threshold for bacterial species delineation is 95–96%.

"The pairwise ANI values are presented in Table S1 (Supplementary Material).".
